# Life history traits and life table analysis of *Lobiopa insularis* (Coleoptera: Nitidulidae) fed on strawberry

**DOI:** 10.1371/journal.pone.0180093

**Published:** 2017-07-11

**Authors:** Nancy Greco, Nicolás Cluigt, Andrew Cline, Gerardo Liljesthröm

**Affiliations:** 1 Centro de Estudios Parasitológicos y de Vectores (CEPAVE), Facultad de Ciencias Naturales y Museo, UNLP, CONICET, La Plata, Argentina; 2 Comisión de Investigaciones Científicas de la Provincia de Buenos Aires, La Plata, Argentina; 3 Plant Pest Diagnostics Branch, California Department of Food & Agriculture, Sacramento, California, United States of America; Ghent University, BELGIUM

## Abstract

*Lobiopa insularis* is a newly reported pest of strawberry in Argentina. We investigated characteristics of its biology in the laboratory, including survivorship and reproduction. We also estimated population growth for *L*. *insularis* fed ripe strawberry fruits. *Lobiopa insularis* was not observed ovipositing on strawberry fruits. A higher proportion of egg masses were recorded from a depth of 1 cm within the soil than on either the soil surface or deeper than 1cm (i.e. between 1and 2 cm) within the soil. The duration of preimaginal developmental stages represented ~18.5% of the total life cycle, while the adult stage represented 81.5%. Survival from egg to adult was 64.20% and mean longevity of females and males adults was 121.84, (SE = 8.86) and 118.58 (SE = 5.90) days, respectively. Females laid eggs only when they were with a male, so reproductive period was dependent on male presence. The number of eggs/female/day was 18.01 (SE = 1.71); and total fecundity was 1655 (ES = 249.53) eggs/female. The long life span of adults and high reproductive output, i.e high fecundity and long reproductive period, indicate that availability and concentration of suitable developmental resources are important factors in the population dynamics of *Lobiopa insularis* associated with strawberry crops.

## Introduction

The life history of an organism refers to the pattern of growth, resource accumulation, differentiation and reproduction exhibited on average by a given species during the sequence of events that occur during its lifetime: e.g. birth, growth during pre-reproductive and reproductive periods [1]. Characteristics of the life cycle and life history of a species greatly influence its population dynamics [2, 3] and are also influenced by the availability of resources which vary significantly over time [4–7]. These factors, coupled with the abilities of polyphagous insects to disperse and use richer habitat patches can lead to pest outbreaks [8–10]. The schedules of survival and age specific reproduction, i.e. life table data, provide insights into the process of population growth [11, 12] and pest population dynamics.

*Lobiopa insularis* (Castelnau) (Coleoptera: Nitidulidae) is a polyphagous species, whose life cycle comprises the egg, three larval instars, pupal and adult stages. When full grown, the larvae fall to the ground and bury themselves to pupate [13]. The adults can disperse long distances and overwinter [14].

Adults can be strongly attracted to ripe fruits in agricultural settings such as peaches, blueberries, raspberries, strawberries, pineapples, apples, melons, tomatoes, corn, stored corn, and dried fruit products, settings where they feed and reproduce. The host range may include small fruits [14, 15]. In natural ecosystems, species of *Lobiopa* are often encountered in subcortical spaces of dead or decaying trees, sap flows, fermenting fruit and flower falls, and occasionally in decomposing leaf litter and debris ([16–21], Cline pers. obs.). This species has also been reported from honeybee hives in North America [22]. The genus *Lobiopa* was recently reviewed [18], which included an overview of the inclusive species and their respective biologies and distributions.

*Lobiopa insularis* has been known as a strawberry pest in USA [14, 23], Brazil [24] and Argentina [13]. In addition to direct feeding damage to the fruit by adults and larvae, they also serve as fungal dispersal agents, increasing yield losses [25–28]. In these countries, current control of *L*. *insularis* in strawberry crops is mainly based on cultural practices. Specifically, techniques include the harvest of strawberries and other fruits in the field immediately upon ripening, and removing damaged, diseased and overripe fruits [14].

Herein, we characterize oviposition traits, analyze survivorship and reproduction, and estimate population growth of *L*. *insularis* fed ripe strawberry fruits.

## Materials and methods

*Lobiopa insularis* colonies were established in the laboratory starting with 16 males and 16 females placed in a plastic container (250 ml) with moistened filter paper in the bottom for oviposition, and fed ripe strawberries. All individuals were collected during October 2007 from commercial strawberry plots in Buenos Aires, Argentina. Several specimens were vouchered at the California State Collection of Arthropods and at the Museum of Natural Sciences of La Plata (La Plata National University, Argentina). The material was labelled as voucher material for this project.

Colonies and experiments were performed under controlled conditions (25 ±1°C, 60–70% RH and 14:10 h L-D).

A trial was performed to assess oviposition preference sites. The experimental unit was a glass container (35 cm length, 30 cm height, 3 cm width) (4 replicates). The containers were filled with sterilized moist soil with two intact ripe strawberries added to the soil surface. Five pairs of adults were introduced to each unit. The number of egg masses and number of eggs per mass were recorded by site for the following: a) on strawberry fruit, b) on soil surface, c) at a depth of 1 cm in soil (just below the soil surface down to a depth of 1 cm), and d) in soil deeper than 1 cm. Observations were made after three days. The proportion of egg masses and the number of eggs per egg mass for each site were analyzed by ANOVA.

To assess survivorship, reproduction, and population growth, three cohorts of 142, 81 and 94 eggs (less than one day old) were individually positioned in plastic containers with ripe strawberries and sterilized soil. The development of the larvae and pupae as well as the adult emergence were recorded.

The duration of developmental stages was estimated and female and male longevity among cohorts was compared by one way ANOVA. If no differences were determined, one way ANOVA was performed to compare longevity between both sexes using individuals of all cohorts.

For each cohort, at the beginning of interval x (each interval lasted one day) we recorded the number of individuals alive, N_(x)_, and their developmental stage (S1 Data). Once in the adult stage the sex was determined according to [29]. This procedure involved observation of the VIII abdominal segment (the so-called “anal sclerite”), which is heavily sclerotized and externally visible in males. The Kaplan-Meier product-limit survival curve analysis [30] was used to examine survival curves. Survival curves of females and males of the three cohorts were compared using a multiple comparison Chi-square test. Comparisons between female and male curves were then tested by the Gehan-Wilcoxon test [31].

Fecundity and fertility were obtained from 25 pairs of newly hatched adults that were kept in individual containers. The number of eggs was counted daily and the daily rate of oviposition calculated as the number of eggs/female/day. Fertility was calculated as the total number of emerged larvae/the total number of eggs and expressed as a percentage.

When the male of a pair died before the female, the female was sustained until death without a male replacement (S1 Data). For each pair, we recorded the following: duration of pre-oviposition period, number of eggs oviposited daily by each female, total number of eggs deposited during a female’s lifetime, day of first oviposition (α), day of last oviposition (ω), duration of reproductive interval (ω – α), and in cases when the male died before the female we also recorded the number of eggs oviposited daily by the female after the death of the male.

We estimated l_x_ and m_x_ distributions calculated for age (x) expressed in days following standard procedures and considering that reproduction typically occurs in the middle of the interval (x, x+1). The corresponding survival to the midpoint of an age interval, the pivotal age, was calculated as: L_x_ = (L_x_+ L_x+1_)/2 [2, 12]. We estimated l_x_ and m_x_ (S1 Table) distributions calculated for pivotal age (x) expressed in days following standard procedures [2]. The following statistics were also calculated based on standard procedure [12]: reproductive rate $Ro=\;\sum_{x=0}^{x=\infty }L_{x}m_{x}$; mean generation time $T=\frac{\left(\sum_{x=0}^{x=\infty }xL_{x}m_{x}\right)}{Ro}$; intrinsic rate of natural increase $r=\sum_{x=0}^{x=\infty }e^{-rx}L_{x}m_{x}$ and reproductive value $V_{x}=\left(\sum_{x=0}^{x=\infty }e^{-rx}L_{x}m_{x}\right)/L_{x}e^{-rx}$.

## Results and discussion

*Lobiopa insularis* was not observed ovipositing on strawberry fruits. The proportion of egg masses was higher at a depth of one cm in soil (0.55, SE: 0.05) than on soil surface (0.32, SE: 0.04) and deeper than one cm (0.19, SE: 0.02) (F = 19.549; df = 2, 9; p<0.001). The number of eggs per egg mass was similar among the soil depths (on soil surface: 13.53, SE: 0.90; at a depth of one cm in soil: 13.60, SE: 0.61 and deeper than one cm: 13.31, SE: 0.02) (F = 0.025; df = 2, 76; p = 0.976). No egg masses were recorded more than 2 cm deep. This may reflect the non-specialized ovipositor found in members of this genus. Some nitidulids, such as *Pocadius* [32] and *Neohebascus* [33] within Nitidulinae, have specialized egg laying apparati due to the specialized hosts that they utilize. However, *Lobiopa*, much like other related nitiduline genera (i.e. *Lasiodactylus* and *Stelidota*) [16, 34] and non-related genera in disparate subfamilies possess a more generalized ovipositor for laying eggs directly on a substrate.

The duration of preimaginal developmental stages represented 18.5% of the duration of the entire life cycle, whereas the adult stage represented 81.5% (Table 1).

**Table 1:** Mean and standard deviation of duration (days) of developmental stages of *Lobiopa insularis* fed ripe strawberry fruits.

	Egg	Instar 1	Instar 2	Instar 3	Pupa	Adult
Cohort 1	4.56(0.92) n = 123	1.19(0.52) n = 123	1.28(0.45) n = 123	13.27(1.65) n = 123	7.58(1.53) n = 120	117.31(62.97) n = 91
Cohort 2	4.36(0.79) n = 68	1.14(0.42) n = 65	1.24(0.43) n = 65	12.73(1.86) n = 65	7.76(1.43) n = 55	119.12(64.82) n = 55
Cohort 3	4.87(1.11) n = 80	1.25(0.59) n = 78	1.20(0.40) n = 78	13.30(1.68) n = 78	7.61(1.50) n = 58	1129.81(64.12) n = 57
Mean	4.60(0.94) n = 271	1.19(0.51) n = 266	1.24(0.43) n = 266	13.10(1.73) n = 266	7.65(1.49) n = 233	122.08(63.97) n = 203

Development was shorter than reported by [35], when the larvae were fed an artificial diet.

Mean longevity of adult females was 121.84, SE = 8.86 and was similar in all cohorts (F = 0.44; df = 2, 121; P = 0.64) while mean longevity of adult males was 118.58, SE = 5.90 and as above was similar in all cohorts (F = 0.32; df = 2, 107; P = 0.72). Mean longevity did not differ between both sexes (F = 0.15; df = 1, 132; P = 0.69). Maximum adult longevity was 219 days for females and 211 for males. These values are lower than those observed by [35] for *L*. *insularis* fed on an artifitial diet: 306 days. Although lower than in this species, high longevity values were also observed in other nitidulids, i.e. *Carpophilus lugubris*: 101.3 and 115.2 days for females and males respectively [36], *Stelidota ferruginea*: 123 days for females [37] and *Stelidota octomaculata*: 125 days for females [38].

**Fig 1:**
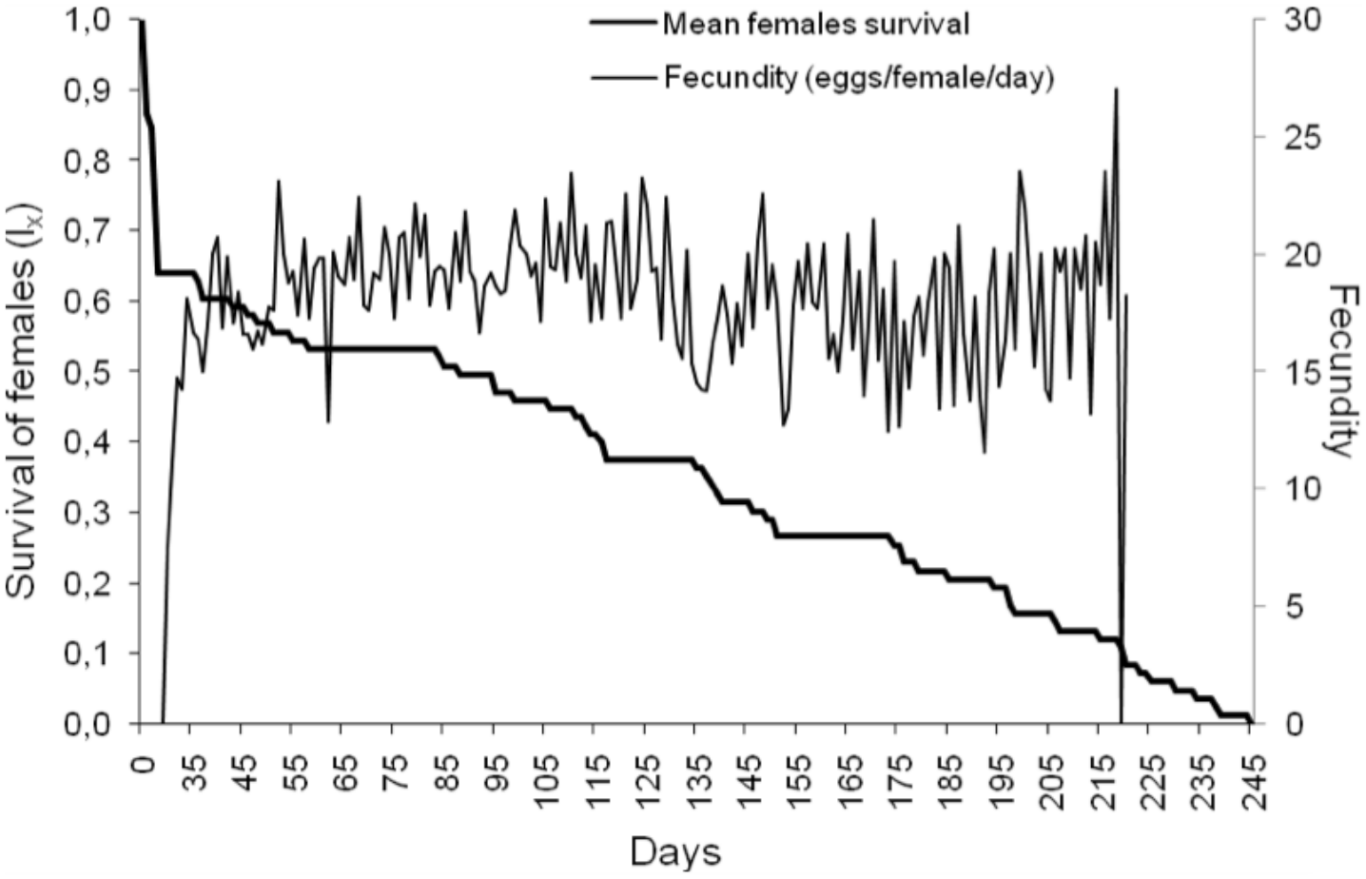
Survival and fecundity of *Lobiopa insularis* fed strawberry.

Female and male survival curves for all cohorts were similar (Chi^2^ = 0.10, df = 3, p = 0.950 and Chi^2^ = 0.88, df = 3, p = 0.644, respectivelly). Survival of females (Fig 1) and males were similar (Gehan-Wilcoxon test statistic = 0.17, p = 0.865). Survival from egg to adult (64.20%) was higher than that obtained by [35] (43.5%).

**Fig 2:**
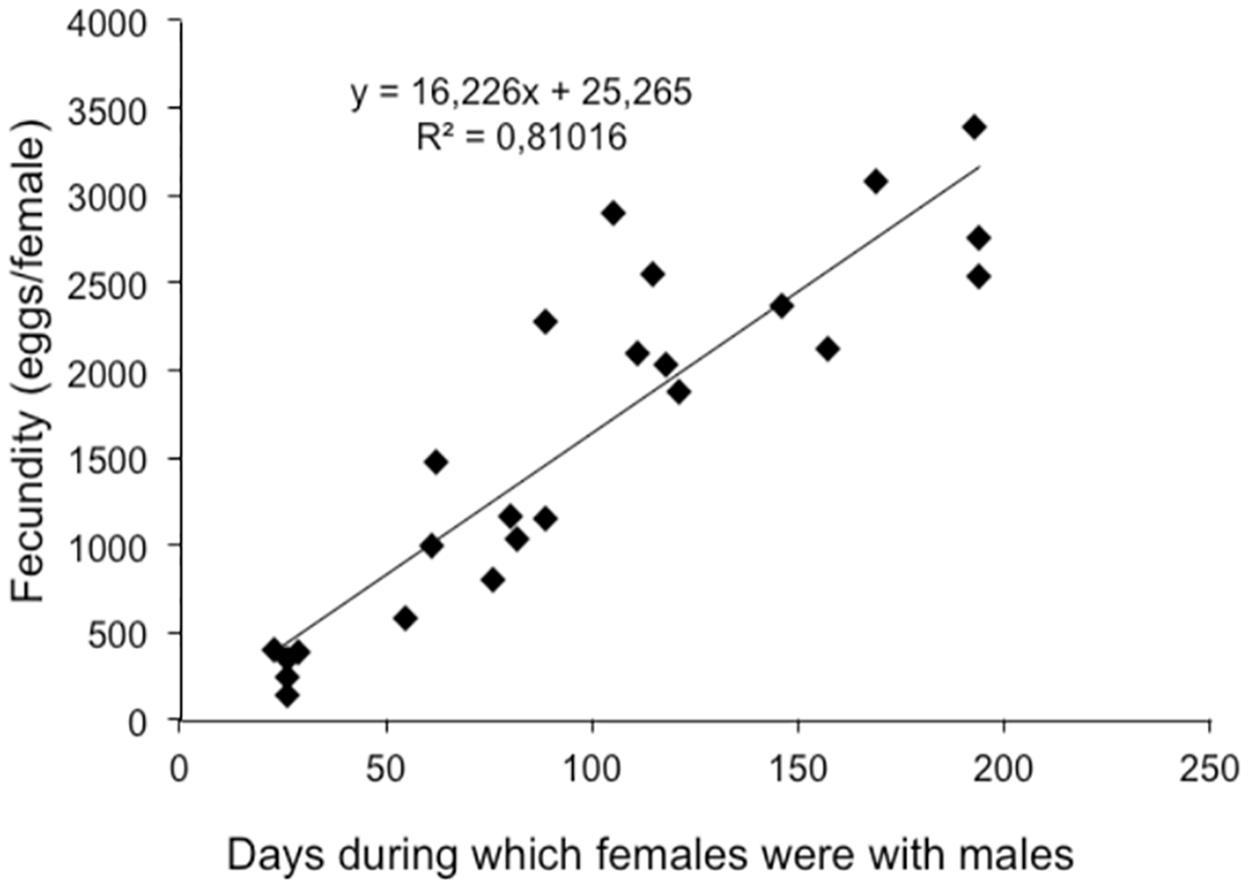
Relationship between fertility and the time lapse during which females were with males.

The number of eggs/female/day (18.1±3.74) was also higher in our study than [35], who reported a daily fecundity of 13.9±5.8 eggs/female/day. The pre-reproductive period was shorter than the reproductive and post-reproductive periods, which were significantly longer and more variable than the former (Table 2). The reproductive period and the fecundity (number of eggs per female) were dependent on the presence of males (Fig 2). The isolation of pairs in this study demonstrated that *L*. *insularis* females laid their eggs only when they were in the presence of a male. Therefore, mating would be essential for oviposition.

**Table 2:** Reproductive attributes of *Lobiopa insularis* fed ripe strawberry fruits.

Attribute	Mean	SE
Pre-reproductive period (days)	6.20	0.26
Age of first oviposition, α (day)	7.20	0.33
Age of last oviposition, ω (day)	97.76	11.26
Reproductive period (days)	91.44	11.17
Post-reproductive period (days)	21.88	5.87
Number of oviposition days (days)	87.44	10.97
Proportion of reproductive period with oviposition	0.82	0.03
Intervals without oviposition (number)	3.20	0.54
Duration of intervals without oviposition (days)	1.27	0.10
Number of eggs/day/female	18.01	1.71
Number of oviposition days after male death	1.90	0.28
Eggs/female	1655.35	249.53

**Fig 3:**
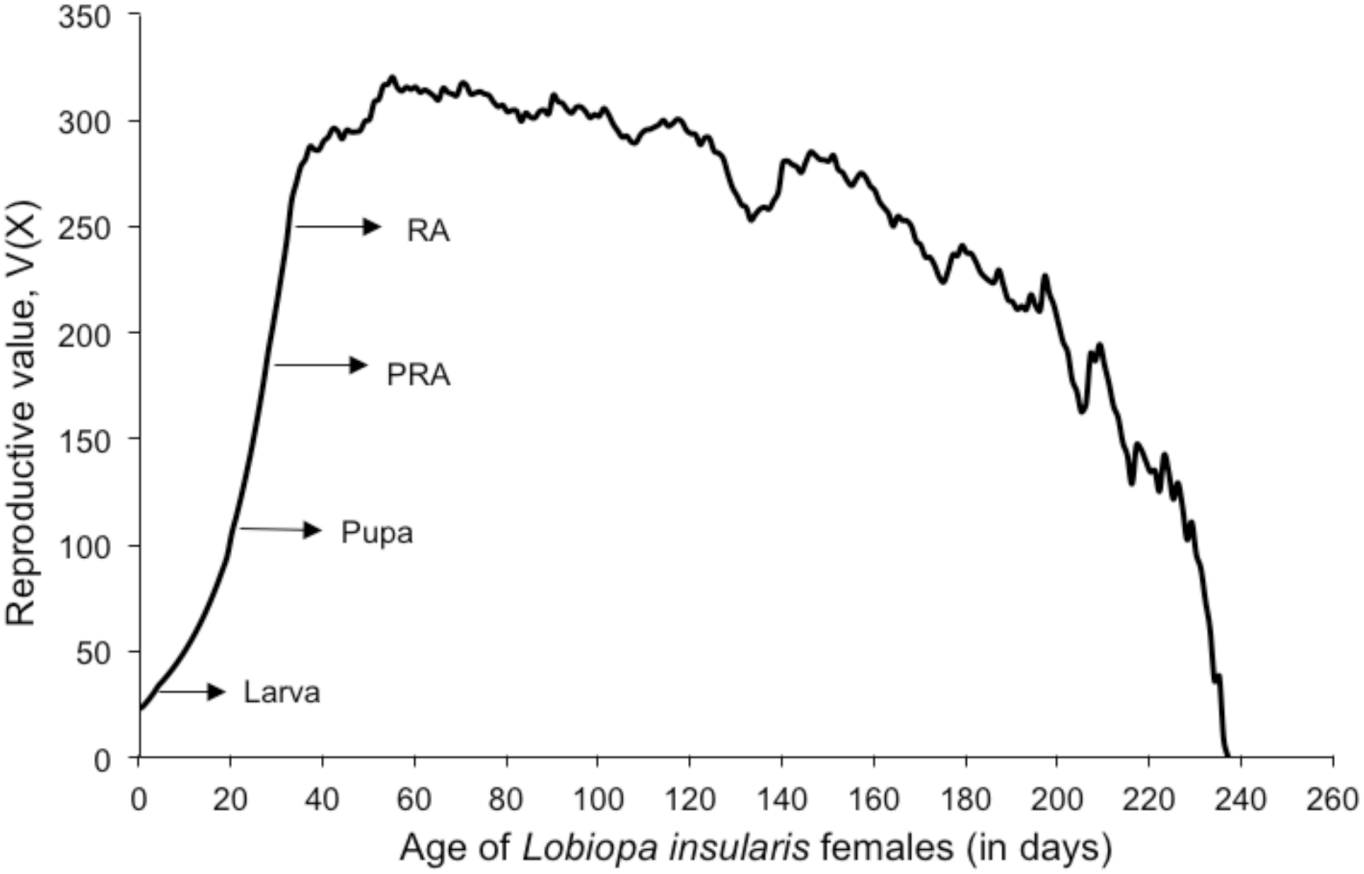
Reproductive value for different ages of *Lobiopa insularis* females (in days) fed ripe strawberry fruits. Arrows indicate the onset of the larval, pupal and adult developmental stages. The pre-reproductive stage (PRA) was differentiated from the onset of the reproductive stage (RA).

Natural selection favors individuals that make the highest proportionate contribution to the population to which they belong, and one measure of an individual’s contribution is it’s reproductive value, V(x) [1]. This measure provides a basis for estimating the present and future contribution of a female age (x) to the population growth rate, r [12]. As is common in other insects, *L*. *insularis* exhibited the greatest V(x) value in the adult stage, however not at the onset of reproduction (age x = α) but rather between ages x = 53 (corresponding to day 26 in the adult stage) to x = 60 (corresponding to day 33 in the adult stage). This time is effectively 20 days after the onset of the reproductive period (Fig 3).

The long life span of adults and high reproductive attributes, including high fecundity and lengthy reproductive period (Table 2), are features of other nitidulid life cycles [14, 36, 37]. The longevity exhibited by adults together in conjunction with a long oviposition period indicates a broad overlap of successive generations during the year [14, 37]. Species that are subject to uncertain environments frequently exhibit extended longevity. Long life span as an adaptation for increasing fitness in uncertain environments is supported by empirical evidence in other groups [39] as well as by mathematical models [40, 41]. At least some individuals must be capable of surviving long, unpredictable periods of unfavorable conditions to ensure population replacement. Species that must find food sources that are scarce and/or widely dispersed tend to be long lived [42]. An overlap of successive generations may be advantageous in subcortical habitats where these beetles typically occur. These microhabitats provide a type of “refugia” wherein larval and pupal stages may have adequate protection from predators and parasitoids as well as non-ephemeral fungal food sources when conditions are optimal. Therefore, overlapping generations may be possible and advantageous. Other subcortical mycophagous cucujoid beetles have expressed this type of reproductive strategy.

Females laid eggs only in the presence of a male, which may indicate that females would have little sperm accumulation capacity within the spermatheca during mating. This characteristic could negatively affect the rate of population increase (Table 3) in natural settings. Insect natality usually is higher at intermediate population densities than at low or high densities. At low densities, difficulties in attracting mates may limit mating [43]. Clearly, if unmated females must find a mate to reproduce after finding a habitable patch, their value as founders is negligible.

**Table 3:** Population parameters of *Lobiopa insularis* fed ripe strawberry fruits.

	Cohort 1 (n = 142)	Cohort 2 (n = 81)	Cohort 3 (n = 94)	Mean	SD
T	102.399	110.123	114.007	108.843	5.909
r	0.062	0.059	0.058	0.060	0.002
R_0_	570.127	663.985	780.644	671.585	105.464

Different mechanisms ensure breeding at a site of colonization such as long-distance attraction via pheromones, or through males accompanying females via phoretic or mating swarms [43]. The concentration of a food resource when the strawberry crop harvest is not done with proper cultural practices attracts *L*. *insularis* adults and thereby increases the rate of meetings between sexes and consequently affects female fecundity. The high fecundity coupled with a long period of resource availability (i.e.the harvest period lasts approximately four months) would lead to high local population increase of this species in strawberry fields. The scarcity of parasitoids, possibly due to recent colonization of strawberry crops or parasitoid satiation, could enhance the pest population increase. The encyrtid *Zeteticontus insularis* (Howard) was cited by [44] as parasitizing *L*. *insularis*, however it has never been registered for strawberry crops in Argentina. In the USA and Brasil, the only parasitoid registered on *L*. *insularis* is the larval parasitoid *Brachyserphus abruptus* (Hymenoptera: Proctotrupidae) [14, 45, 46].

Availability and concentration of resources appear to be the most important factors affecting the population dynamics of *Lobiopa insularis* associated with strawberry crops. However, soil predators (e.g. staphylinids and carabids) [47] may deserve more attention as potential control agents for eggs and pupae that are buried in the soil.

## Supporting information

S1 Data Daily survival and oviposition data of *L*. *insularis* fed on strawberry in laboratory Greco et al. (XLSX)
